# Efficacy and safety of body weight-adapted oral cholecalciferol substitution in dialysis patients with vitamin D deficiency

**DOI:** 10.1186/s12882-015-0116-3

**Published:** 2015-08-04

**Authors:** Emanuel Zitt, Hannelore Sprenger-Mähr, Michael Mündle, Karl Lhotta

**Affiliations:** Department of Nephrology and Dialysis, Academic Teaching Hospital Feldkirch, Carinagasse 47, A-6800 Feldkirch, Austria; Vorarlberg Institute for Vascular Investigation and Treatment (VIVIT), Academic Teaching Hospital Feldkirch, Feldkirch, Austria

**Keywords:** Chronic dialysis, Mineral metabolism, Hyperparathyroidism, Vitamin D

## Abstract

**Background:**

Vitamin D deficiency is highly prevalent in dialysis patients. Whether substitution of native vitamin D in these patients is beneficial is a matter of ongoing discussion, as is the optimal dosing schedule. The purpose of this study was to investigate the efficacy and safety of a body-weight adapted oral dosing regimen of cholecalciferol in dialysis patients.

**Methods:**

In a prospective single-center study 56 prevalent dialysis patients with a baseline 25OHD_3_ level <20 ng/mL received 100 IU of cholecalciferol per kg body weight once weekly orally for 26 weeks. 25OHD_3_ was measured at baseline and at study end, iPTH every three months, serum calcium and phosphorous monthly. Concurrent medication including phosphate binders, calcitriol and cinacalcet and dialysate calcium concentration remained unchanged throughout the study.

**Results:**

Baseline 25OHD_3_ was 9.9 ± 4.1 ng/mL and increased to 26.1 ± 8.8 ng/mL (P = 0.01). Fourteen patients (27 %) achieved a level >30 ng/mL and all others above 20 ng/mL. Cinacalcet therapy was positively associated with the increase in 25OHD_3_ (P = 0.024). The plasma iPTH level significantly decreased from median 362 pg/mL to 297 pg/mL (P = 0.01). This decline was more pronounced in patients with higher baseline iPTH levels (P < 0.01) and differed significantly dependent on concurrent calcitriol therapy. A significant iPTH decrease was observed in patients receiving calcitriol (P = 0.031). Serum calcium and phosphorous did not change significantly throughout the study period. Cholecalciferol substitution was well tolerated without adverse effects.

**Conclusion:**

The dosing regimen of oral cholecalciferol supplementation with 100 IU per kg body weight per week for 26 weeks in dialysis patients with vitamin D deficiency causes a significant increase in 25OHD_3_ close to the supposed target level of 30 ng/mL and a significant reduction in iPTH, without affecting serum calcium or phosphorous levels.

## Background

Vitamin D deficiency affects about one billion people worldwide. It is especially prevalent at higher altitudes, where sun exposure is low [1]. In Germany, 58 % of men and 57 % of women have 25-hydroxy-vitamin D3 (25OHD_3_) levels below 20 ng/mL, the most current definition of vitamin D deficiency [2]. Vitamin D deficiency has been found to be associated with a multitude of diseases such as cancer, cardiovascular disease, diabetes and autoimmunity [1]. A recent umbrella review of systematic reviews and meta-analyses of observational studies and randomized trials comes to the conclusion that among 137 outcomes vitamin D supplementation affects only birth weight, dental caries in children and parathyroid hormone (PTH) concentrations in patients with chronic kidney disease [3]. The authors conclude that current evidence suggests vitamin D supplementation only for pregnant women, children and for CKD patients with and without dialysis with the goal of lowering PTH levels. The results of this umbrella analysis, however, are based on a meta-analysis of active vitamin D compounds in dialysis patients [4]. With regard to substitution of native vitamin D in CKD patients, such data are not available, because no large randomized controlled trials have been performed. Nevertheless, recommendations issued by NKF KDOQI, KDIGO and the European Best Practice Group guidelines, support correction of vitamin D deficiency in CKD patients [5–7]. However, only the KDIGO guidelines suggest supplementation in dialysis patients [6]. Concerning dose, treatment strategies used for the general population are recommended. Most studies addressing treatment of vitamin D deficiency in dialysis patients use weekly doses of 50.000 IU ergocalciferol or cholecalciferol, as recommended by KDOQI for the general population and for patients with CKD stage 3 to 4 [1, 5]. The lowest weekly doses reported in the literature in hemodialysis patients are 4500 IU (640 IU daily), 10.000 IU (40.000 IU monthly) and 10.333 IU cholecalciferol [8–10]. At a consensus meeting held in August 2012 experts from Germany, Austria and Switzerland recommended treatment of vitamin D deficiency with 70 to 140 IU cholecalciferol per kilogram body weight per week to increase 25OHD_3_ levels by 10 ng/mL (Pharma Report, Der Internist, 54(2), 2013). These recommendations were recently confirmed in a systematic review of vitamin D supplementation, body weight and serum 25OHD_3_ response [11].

The aim of the present study was to determine the efficacy of a dosing regimen of 100 IU of cholecalciferol per kg per week, over a period of six months in prevalent dialysis patients and to describe its effects on serum calcium, serum phosphorous and plasma iPTH.

## Methods

We prospectively included prevalent adult hemodialysis and peritoneal dialysis patients with a serum 25OHD_3_ level <20 ng/mL as determined in December 2012. Individuals with a serum calcium >2.55 mmol/L or serum phosphorous >2.50 mmol/lL, or patients unable to give informed consent were excluded. This was a single-center study performed at the Academic Teaching Hospital Feldkirch in Feldkirch (Austria) at latitude 47.23° North. Patients were given oral cholecalciferol (Oleovit D3 Tropfen®, Fresenius Kabi, Graz, Austria, 400 IU per drop) 100 IU per kg body weight once weekly. Treatment was started in June 2013 and continued for 26 weeks until December 2013. Hemodialysis patients received the droplets at the end of the first dialysis section of the week. Peritoneal dialysis patients were advised to take the prescribed dose once per week at home. The dose of phosphate binders, calcitriol and cinacalcet was held constant during the study period. The dialysate calcium concentration was 1.25 mmol/L in all patients and was kept unchanged throughout the study. Retrospectively, 25OHD_3_ levels measured in December 2011 were collected and compared to evaluate the random variability of 25OHD_3_ levels without cholecalciferol substitution (December 2011 to December 2012).

The following demographic, anthropometric, clinical and medical parameters were collected at study entry: age, sex, height, body weight (dry weight), renal disease, treatment and dose of calcitriol, cinacalcet, and phosphate binders. Serum calcium and phosphorous were measured monthly, plasma iPTH every three months. The investigation was registered with AGES (Austrian Agency for Health and Food Safety, registration number NIS002983) as a non-interventional study without any interventions or investigations beyond routine patient management. Single center non-interventional studies do not require ethical approval by Austrian law. The study was performed in agreement with the Declaration of Helsinki and Austrian law. All participating patients gave written informed consent to participate in the study.

### Statistical analysis

Continuous data are presented as mean ± SD or median (25^th^, 75^th^ percentile) dependent on the distribution of the variables, categorical variables as absolute and relative frequencies (%). Normal distribution was evaluated with the Kolmogorov-Smirnov test. Levels of 25OHD_3_, iPTH, calcium and phosphorous before and after cholecalciferol supplementation were compared using the paired T test (Ca, P) or the Wilcoxon rank sum test (iPTH). Group differences were determined by ANOVA for continuous variables and by chi-square test or Fisher exact test for categorical variables as appropriate. Multiple linear regression models were applied to assess predictors of 25OHD_3_ and iPTH response with cholecalciferol supplementation. A *P* value less than 0.05 (two-sided) was deemed to indicate statistical significance. All statistical analyses were performed with IBM SPSS Statistics, version 22 (SPSS Inc., Chicago, IL).

## Results

**Fig. 1:**
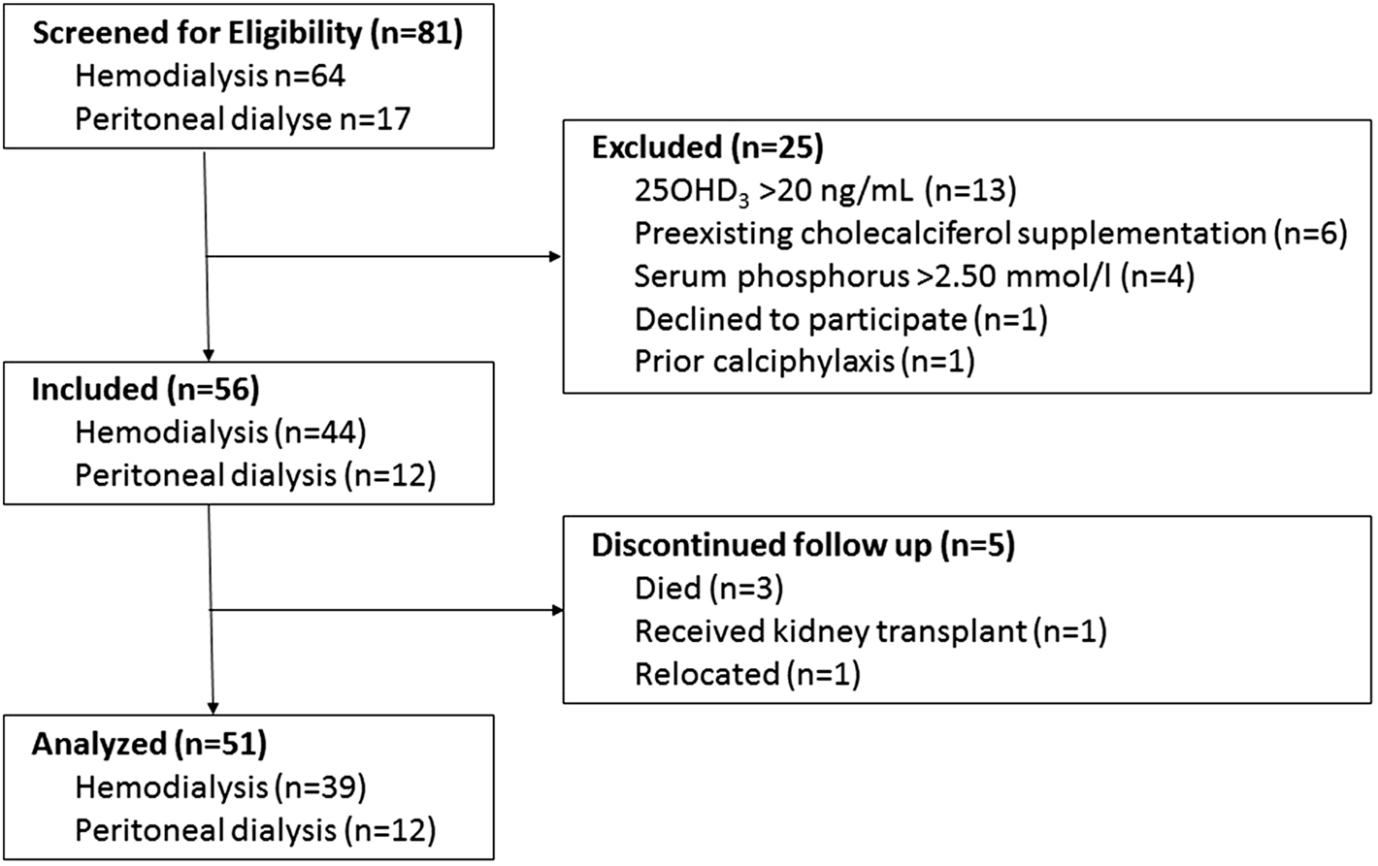
Patient flow diagram

Fifty-six patients, 44 on hemodialysis and 12 on peritoneal dialysis, with a mean age of 63 ± 18 years were included out of 82 screened patients. Of those entering the study 29 (52 %) were men and 27 (48 %) were women. Fifty-one patients (91 %) completed the study. Three patients died during the study, one received a kidney transplant and one moved to another dialysis unit. Causes of death were cardiac arrest, advanced breast cancer and sepsis, none of them suspected to be linked to the cholecalciferol substitution (Fig. 1).

**Fig. 2:**
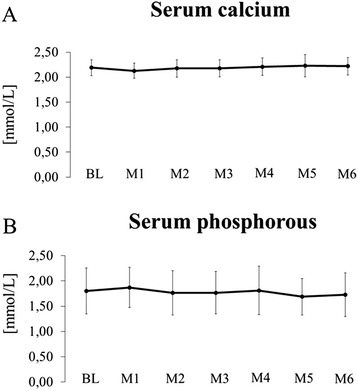
Course of serum calcium (**a**) and phosphorous (**b**) over 26 weeks of cholecalciferol supplementation. BL, baseline; M1-M6, months 1–6

Baseline characteristics of participating patients are listed in Table 1. Results for 25OHD_3,_ serum calcium, phosphorous and plasma iPTH before and after 26 weeks of cholecalciferol substitution are shown in Table 2. Mean 25OHD_3_ had been 9.0 ± 4.1 ng/mL in December 2011 (one year prior to the evaluation of the pre-supplemental concentration) and remained unchanged in December 2012 without substitution (9.9 ± 4.1 ng/mL). Patients received a mean weekly cholecalciferol dose of 7603 ± 1855 IU. After six months of substitution 25OHD_3_ increased to 26.1 ± 8.8 ng/mL (P < 0.01) with a mean increase of 16.2 ± 7.8 ng/mL. At the end of the study 14 (27 %) patients had a 25OHD_3_ level >30 ng/mL, and all 37 (73 %) other patients a level >20 ng/mL. Serum calcium and phosphorous remained constant. Figure 2 shows the course of monthly serum calcium and phosphorous levels during the whole study period. We observed six hypercalcemic episodes (serum calcium >2.55 mmol/L) representing 1.9 % of all monthly measurements (n = 306). Hypercalcemia was noted in four patients (three patients with one episode, one patients with three episodes). A total of 13 episodes (4.2 %) of hyperphosphatemia (serum phosphorus >2.50 mmol/L) occurred during the whole study period in nine patients (five patients with one episode, 4 patients with two episodes). Table 3 shows the results of the multiple linear regression analysis for the 25OHD_3_ increase. Use of cinacalcet (P = 0.024) and hemodialysis versus peritoneal dialysis treatment (P = 0.045) was significantly associated with a better response to cholecalciferol substitution.

**Table 1:** Baseline characteristics of patients

Gender	
Men	29 (52 %)
Women	27 (48 %)
Age (years)	63 ± 18
Body Weight (kg)	76 ± 19
Dialysis vintage (months)	46 (24,63)
Renal disease	
Diabetic nephropathy	16 (29 %)
Hypertensive nephropathy	10 (18 %)
Glomerulonephritis	13 (23 %)
Polycystic kidney disease	4 (7 %)
Interstitial nephritis	1 (2 %)
Other	12 (21 %)
Diabetes mellitus	19 (34 %)
Vascular access	
AV fistula	30 (77 %)
AV graft	5 (13 %)
Catheter	4 (10 %)
Creatinine (mg/dl)	8.2 ± 2.9
Urea (mg/dl)	125 ± 34
Albumin (g/dl)	3.5 ± 0.3
Hemoglobin (g/l)	111 ± 12.6
Calcitriol	40 (71 %)
Dose per day (μg)	0.26 ± 0.10
Treatment vintage (months)	34 (19, 51)
Ca-containing PB	20 (36 %)
Treatment vintage (months)	29.5 (8.3, 57.5)
Ca-free PB	24 (43 %)
Treatment vintage (months)	30.5 (19.5, 48.5)
Cinacalcet	26 (46 %)
Dose per day (mg)	52 ± 26
Treatment vintage (months)	33 (15.5, 57.5)

Abbreviations: Ca, calcium; PB, phosphate binder. Data are presented as mean ± SD or median (25^th^ percentile, 75^th^ percentile)

**Table 2:** 25OHD_3,_ calcium, phosphorous and iPTH before and after cholecalciferol substitution

	Baseline	After 26 weeks	P value
25OHD_3_ (ng/mL)	9.9 ± 4.1	26.1 ± 8.8	<0.01
Calcium (mmol/L)	2.20 ± 0.16	2.22 ± 0.17	0.45
Phosphate (mmol/L)	1.80 ± 0.45	1.73 ± 043	0.31
iPTH (pg/mL)^a^	362 (258, 498)	297 (202, 434)	0.01

^a^median (25^th^, 75^th^ percentile)

**Table 3:** Multiple linear regression analysis for 25OHD_3_ increase (ng/mL) with cholecalciferol supplementation

Parameter	Β (95 % CI)	P
Age (years)	0.06 (−0.06, 0.17)	0.347
Male gender (vs female)	3.30 (−0.82, 7.42)	0.113
Dialysis mode		0.045
Peritoneal dialysis	−5.01 (−9.89, −0.12)	
Hemodialysis	Ref.	
Calcitriol therapy		0.637
Yes	1.07 (−3.46, 5.59)	
No	Ref.	
Cinacalcet therapy		0.024
Yes	4.89 (0.66, 9.13)	
No	Ref.	

Covariates in the model included: age, gender, dialysis mode, calcitriol therapy, cinacalcet therapy

**Fig. 3:**
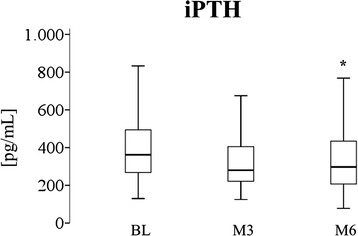
Course of plasma iPTH over 26 weeks of cholecalciferol supplementation. BL, baseline; M3, month 3; M6, month 6. iPTH decreased significantly from 362 to 297 pg/mL (P = 0.01)^*^ after 26 weeks of cholecalciferol substitution

**Fig. 4:**
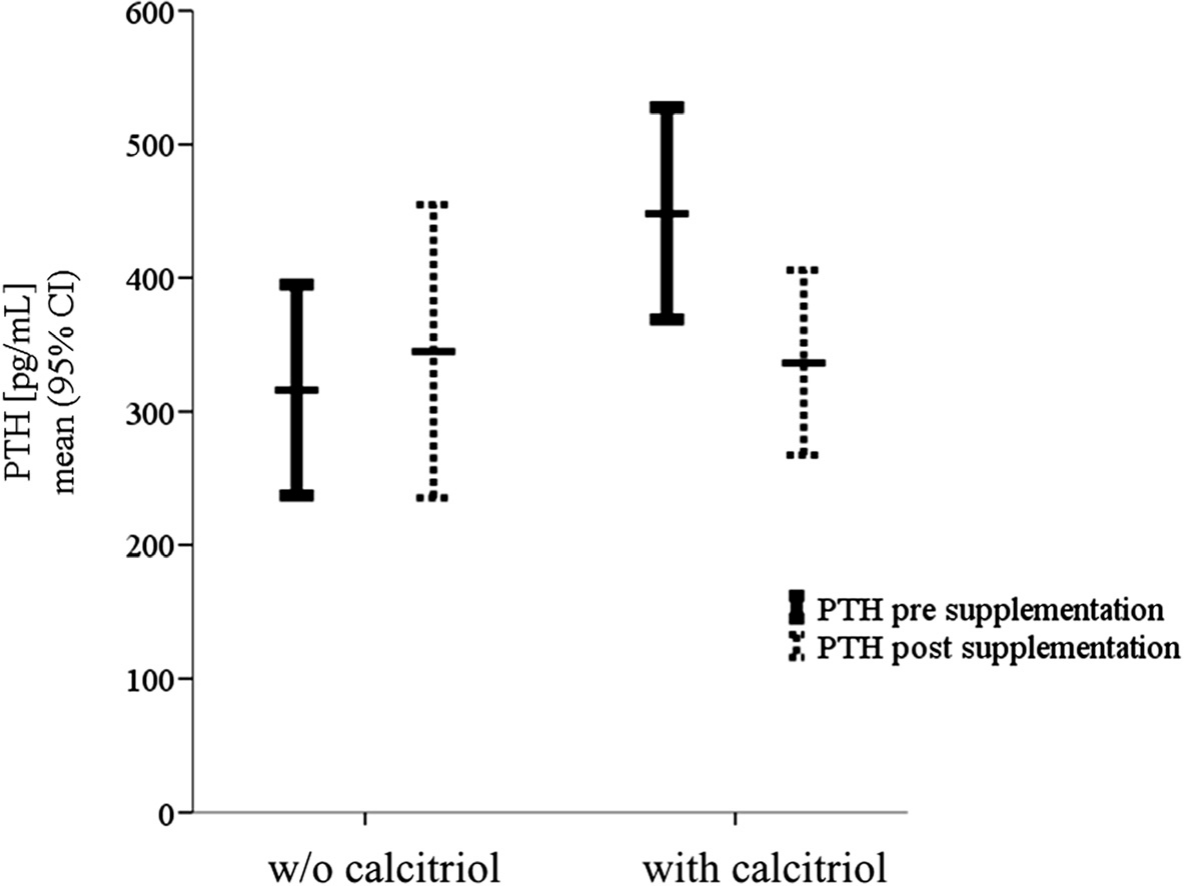
iPTH course after six months of cholecalciferol supplementation in patients with and without (w/o) calcitriol. In patients without calcitriol iPTH did not change significantly (iPTH post 345 ± 190 *vs* iPTH pre 316 ± 137 pg/mL). Patients with calcitriol showed a significant iPTH reduction (336 ± 207 *vs* 448 ± 238 pg/mL, p = 0.031)

As shown in Fig. 3, iPTH levels decreased from median 362 pg/mL (258, 498) to 297 (202, 434) pg/mL (P = 0.01) during the treatment period. The only significant predictor of iPTH reduction was baseline iPTH with a greater decrease observed in patients with higher baseline values (Table 4). Patients who were under calcitriol treatment showed a greater iPTH response to cholecalciferol as compared to those without calcitriol (Fig. 4).

**Table 4:** Multiple linear regression analysis for ΔiPTH (iPTH _post_-iPTH _pre_) (pg/mL) with cholecalciferol supplementation

Parameter	Β (95 % CI)	P
Age (years)	−2.41 (−5.18, 0.37)	0.088
iPTH pre (pg/mL)	−0.56 (−0.80, −0.32)	<0.001
Δ25(OH)vitamin D (ng/mL)	−3.61 (−10.65, 3.42)	0.307
Calcitriol		0.215
Yes	−70.39 (−183.22, 42.45)	
No	Ref.	
Cinacalcet		0.483
Yes	37.59 (−69.38, 144.56)	
No	Ref.	

Abbreviations: iPTH_pre_, iPTH level before cholecalciferol substitution; iPTH_post_, iPTH level after cholecalciferol substitution; Δ25(OH)vitamin D, absolute change in 25(OH)D_3_ with cholecalciferol substitution. Covariates in the model included: age, baseline iPTH, absolute change in 25(OH)D, calcitriol therapy, cinacalcet therapy

## Discussion

Whether individuals with and without kidney disease suffering from vitamin D deficiency need vitamin D substitution and how this substitution should be given are matters of debate. When determining the optimal vitamin D dose it is crucial to understand the kinetics of conversion of vitamin D to 25OHD_3_. At present, the exact liver enzyme or enzymes for 25-hydroxylation are still unknown, but the most likely candidates are CYP2R1, CYP3A4 and CYP27A [12–14].

Heaney described a biphasic pattern of 25-hydroxylation. At low doses of cholecalciferol the compound is completely converted to 25OHD_3_, meaning that cholecalciferol concentrations remain low and 25OHD_3_ serum concentrations rise rapidly [15]. When daily cholecalciferol doses above 2000 IU are given, the enzymes become saturated, serum cholecalciferol rises and some of it is stored in fat tissue, and 25OHD_3_ serum levels continue to rise slowly. The 25OHD_3_ breakpoint of this biphasic response is at about 35 ng/mL, which is practically identical to the level, below which PTH levels begin to rise in response to low 25OHD_3_ levels [16]. This study clearly explains why 25OHD_3_ increases rapidly under low doses of cholecalciferol and that supraphysiologic doses above 2000 UI per day cause an only marginal further increase. Although these results were obtained in healthy individuals, there is no reason to believe that CKD patients would behave differently. In fact, published studies of cholecalciferol substitution in CKD and hemodialysis patients are in line with this observation [9, 10, 17–22].

We are not aware of any study in dialysis patients investigating a body weight-based oral cholecalciferol dosing regimen. Body weight is the most important determinant of the 25OHD_3_ response to a given cholecalciferol dose [11, 23]. This phenomenon may be partly explained by the fact that in patients with higher body weight and more body fat an increasing proportion of administered vitamin D is stored in fat tissue and not available for 25-hydroxylation [24]. Earlier studies with oral vitamin D2 (ergocalciferol) supplementation have shown an inverse correlation between peak serum vitamin D2 levels and body-mass-index or obesity, respectively [24] . In older (≥64 years) healthy persons, but not in younger adults (20–40 years) a significant negative association between baseline 25OHD_3_ and BMI or adiposity was found. When these persons were supplemented in a randomized placebo-controlled trial (cholecalciferol 600 IU/day vs placebo), the 25OHD_3_ response to supplemental cholecalciferol was negatively associated with baseline BMI (the mean adjusted change in 25OHD_3_ decreased by approximately 2.6 ng/mL with every 5 kg/m^2^ increase in BMI) [25]. Therefore, we believe that the body-weight adapted supplementation should not be adjusted to lean body weight and might possibly be even more precise when corrected for BMI or fat mass. This very interesting point should be further investigated in future studies, ideally with quantifying the vitamin D content in adipose tissue.

Van Groningen calculated the loading dose of cholecalciferol needed to reach a 25OHD_3_ level of 30 ng/mL using the formula IU = 100 x (30-25OHD_3_) x kg BW. Using this formula, the mean dose for our patients would have been 152.000 IU or a weekly dose of 5850 IU for 26 weeks [23]. In a recent meta-analysis Zittermann et al. showed an exponential dose requirement when trying to achieve higher 25OHD_3_ levels [11]. They found a logarithmic association between cholecalciferol dose per kg body weight and the increment in 25OHD_3._ For a 70-year-old person with a body weight of 75 kg the calculated daily dose to achieve a target of 20 ng/mL was 308 IU and for a target of 30 ng/mL the daily dose was 1460 IU (corresponding to a weekly dose of 10.220 IU). The weekly dose we used based on the recommendation of 100 IU per kg body weight lies between these calculated doses. There are other studies of comparable vitamin D doses in dialysis patients. Armas et al. used a weekly cholecalciferol dose of 10.333 IU in twenty patients for 15 weeks. Serum 25OHD_3_ increased by 23.6 ng/mL [9]. Jean et al. tested a mean daily dose of 640 IU (400–1200 IU) in 149 hemodialysis patients over six months [10]. They found an impressive increase in 25OHD_3_ of 38.4 ng/mL. This favorable result compared to ours and that of Armas et al. may be explained by the use of daily low doses as compared to weekly higher doses. These frequent low doses possibly allow for more quantitative 25-hydroxylation in the liver [15]. These and our study clearly show that these rather low doses are effective for reaching 25OHD_3_ levels at or close to the target range of 30 ng/mL. From these data we do not believe that daily doses in excess of 3.500 IU are necessary to correct vitamin D deficiency in dialysis patients, as recently suggested [26]. Of note, these dose recommendations apply only to cholecalciferol and not to ergocalciferol treatment. As ergocalciferol seems to be less effective in increasing 25OHD_3_ levels, higher doses may be required when using vitamin D_2_ [27]. Although the optimal 25OHD_3_ level for CKD and dialysis patients is unknown, a target of 30 ng/mL may be reasonable for maximal PTH suppression. Using our dosing regimen the mean 25OHD_3_ level was 26 ng/ml, but only a minority of patients achieved the 30 ng/mL goal. Therefore, it may be necessary to increase the weekly dose especially in those patients with very low basal levels [23]. Keeping in mind the ongoing debate about erythropoietin and iron therapy in dialysis patients, we are cautious about recommendations of high and unphysiological cholecalciferol or ergocalciferol doses. It may be wise to use the lowest possible vitamin D dose to achieve a (yet to be clearly defined) 25OHD_3_ target level.

In our study cinacalcet therapy was associated with a higher increase in 25OHD_3_. This association has not been described until now. Cinacalcet has been shown to reduce FGF-23 levels in dialysis patients independently from its effect on iPTH [28–30]. In animal models, FGF-23 induces expression of CYP24A1 or 24-hydroxylase in the liver [31, 32]. It could be speculated that induction of CYP24A1 by FGF-23 would lead to enhanced 24-hydroxylation of 25OHD_3_ to 24,25OHD_3_, the first step in 25OHD_3_ catabolism and therefore contribute to vitamin D deficiency in CKD. However, 24,25OHD_3_ levels have been found to be rather low in this group of patients despite elevated FGF-23 [32–34]. Nevertheless, we speculate that reduction of FGF-23 and consecutive decrease of CYP24A1 activity and 24-hydroxylation can explain our observation. Clearly, further studies are needed to confirm this finding and mechanism in dialysis patients.

The lower response in peritoneal dialysis patients is most likely explained by lower compliance with home-based intake than in hemodialysis patients, who were given their medication at the dialysis ward resulting in a 100 % compliance rate.

A still unresolved question is the optimal 25OHD_3_ target level for CKD and dialysis patients. The recent report from the Institute of Medicine states that a 25OHD_3_ serum level of 16 ng/mL covers the requirements of half the general population and a level of 20 ng/mL of 97.5 % of the population [35]. According to the umbrella review by Theodoratou et al. a lowering of PTH is the only indication for vitamin D substitution in CKD and hemodialysis patients. These data, however, are based on studies using mainly active vitamin D compounds [4, 36]. There were no other health-related effects of vitamin D therapy in this population. In agreement with these findings, a recent small study in hemodialysis patients also showed no positive effects of cholecalciferol substitution on muscle strength, functional capacity, quality of life and pulse wave velocity [37]. Active vitamin D therapy lowers PTH by 19.7 pg/mL (95 % CI 34.3-5.2) in CKD patients not on dialysis and by 78.4 pg/mL (119.4-37.1) in dialysis patients [3]. Since PTH levels increase, at least in the general population, as serum 25OHD_3_ drops below 30 ng/mL, we suggest that this would be the appropriate target level for CKD and dialysis patients. A very recent publication, however, suggests that this level might be too low for CKD stage 3 to 5 patients, and that a maximum effect on PTH requires levels between 42 to 48 ng/mL [38]. The central question is whether, as shown for active vitamin D compound, native vitamin D is also able to lower PTH levels. We observed a significant decline in PTH levels in our patients. A significant decline in PTH has not been uniformly found in studies of vitamin D substitution in CKD patients. In general, patients in studies without a significant PTH response had iPTH levels below 200 pg/mL [9, 39, 40], whereas in studies of a significant PTH decline reported basal levels were above 200 pg/mL [10, 19, 41–43]. This hypothesis is strengthened by our observation that baseline PTH is the best predictor of a PTH decline under cholecalciferol substitution. Based on our results and the published literature it can be concluded that native vitamin D substitution in patients with low 25OHD_3_ levels is able to cause some PTH reduction, albeit not to a degree as seen with active vitamin D compounds. However, the risk of developing hypercalcemia or hyperphosphatemia is lower when using native vitamin D as shown in our study. Recently Armas et al. found an unchanged calcium absorption despite a high cholecalciferol supplementation of 20,000 IU weekly for 12- to 13 weeks [44]. Considering the fact that at present PTH lowering is the only goal for cholecalciferol substitution in dialysis patients, it remains doubtful whether patients with low PTH levels are candidates for native vitamin D substitution at all.

One argument against native vitamin D substitution in dialysis patients has been that most of them already receive activated vitamin D compounds and therefore would not profit from additional vitamin D substitution. Our results, however, suggest that cholecalciferol substitution further decreases PTH in patients already given low doses of calcitriol. Whether this effect is also present in patients receiving calcitriol doses above 0.25 μg per day needs further investigation.

Our study has some limitations and strengths. First, the number of participants is rather small, but still included more than twice the number of dialysis patients with cholecalciferol supplementation compared to recently published randomized controlled trials [9, 37, 40]. Second, the study lacks a control group without cholecalciferol substitution, although the retrospective analysis of the 25OHD_3_ course in the year prior to the cholecalciferol substitution found no change in 25OHD_3_ without substitution. We also did not measure 1,25OHD_3_ levels, which would have allowed us to determine whether these levels were associated with outcomes, especially iPTH levels. In addition, we also did not measure FGF-23. One major strength of this study is that treatment with phosphate binders, active vitamin D compounds and cinacalcet as well as dialysate calcium was kept strictly constant during the whole treatment period. For this reason, we are sure that the observed iPTH changes were only a consequence of cholecalciferol substitution.

## Conclusion

We found that in dialysis patients with vitamin D deficiency oral substitution with cholecalciferol at a body weight-adapted dose of 100 IU per kg body weight leads to a significant increase in 25OHD_3_ levels close to the supposed target range of 30 ng/mL. This therapy also causes a significant drop in iPTH levels, even in patients with preexisting calcitriol therapy. In addition, it is safe without causing a rise in serum calcium or phosphorous.

## Abbreviations

25OHD_3_: 25-hydroxy-vitamin D_3_
iPTH: Intact parathyroid hormone
CKD: Chronic kidney disease
NKF KDOQI: National Kidney Foundation Kidney Disease Outcome Quality Initiative
KDIGO: Kidney Disease Improving Global Outcomes
CYP: Cytochrome P
FGF-23: Fibroblast growth factor-23
